# Enhancing interaction of actin and actin-binding domain 1 of dystrophin with modulators: Toward improved gene therapy for Duchenne muscular dystrophy

**DOI:** 10.1016/j.jbc.2022.102675

**Published:** 2022-11-11

**Authors:** Piyali Guhathakurta, Anna L. Carter, Andrew R. Thompson, Dillon Kurila, Jeffrey LaFrence, Li Zhang, Jake R. Trask, Bri Vanderheyden, Joseph M. Muretta, James M. Ervasti, David D. Thomas

**Affiliations:** Department of Biochemistry, Molecular Biology and Biophysics, University of Minnesota, Minneapolis, Minnesota, USA

**Keywords:** actin, dystrophin, time-resolved FRET, high-throughput screening, actin-binding, AAV, adeno-associated virus, ABD, actin-binding domain, ABP, actin-binding protein, AF-actin, Alexa 568 F-actin, CRC, concentration-response curve, DMD, Duchenne muscular dystrophy, DMSO, dimethyl sulfoxide, FLTPR, fluorescence lifetime plate reader, hDYS, human dystrophin, HTS, high-throughput screening, hUTR, human utrophin, LOPAC, Library of Pharmacologically Active Compounds, TR-FRET, time-resolved FRET

## Abstract

Duchenne muscular dystrophy is a lethal muscle disease, caused by mutations in the gene encoding dystrophin, an actin-binding cytoskeletal protein. Absence of functional dystrophin results in muscle weakness and degeneration, eventually leading to cardiac and respiratory failure. Strategies to replace the missing dystrophin *via* gene therapy have been intensively pursued. However, the dystrophin gene is too large for current gene therapy approaches. Currently available micro-dystrophin constructs lack the actin-binding domain 2 and show decreased actin-binding affinity *in vitro* compared to full-length dystrophin. Thus, increasing the actin-binding affinity of micro-dystrophin, using small molecules, could be a beneficial therapeutic approach. Here, we have developed and validated a novel high-throughput screening (HTS) assay to discover small molecules that increase the binding affinity of dystrophin’s actin-binding domain 1 (ABD1). We engineered a novel FRET biosensor, consisting of the mClover3, fluorescent protein (donor) attached to the C-terminus of dystrophin ABD1, and Alexa Fluor 568 (acceptor) attached to the C-terminal cysteine of actin. We used this biosensor in small-molecule screening, using a unique high-precision, HTS fluorescence lifetime assay, identifying several compounds from an FDA-approved library that significantly increase the binding between actin and ABD1. This HTS assay establishes feasibility for the discovery of small-molecule modulators of the actin–dystrophin interaction, with the ultimate goal of developing therapies for muscular dystrophy.

Duchenne muscular dystrophy (DMD) is a fatal X-linked disorder, caused by one or more mutations in the DMD gene, which encodes dystrophin, an actin-binding cytoskeletal protein (1). Dystrophin provides a structural link between the skeletal muscle cytoskeleton and the extracellular matrix, and it is essential to maintain muscle integrity (2, 3). Dystrophin connects the actin cytoskeleton with the extracellular matrix by binding directly to actin and the sarcolemmal dystroglycan protein complex, which spans the sarcolemma and attaches to extracellular matrix proteins (4). Absence of dystrophin disrupts this critical linkage and decreases sarcolemmal integrity, resulting in the pathological manifestation of DMD. Current therapeutic strategies for DMD mainly rely on two approaches: (1) restoring the expression and/or function of dystrophin using gene-based, cell-based, and protein replacement therapies and (2) improving muscle function and quality by targeting the downstream pathological changes (5, 6).

A major barrier for achieving dystrophin restoration using viral gene therapy is the large size of the dystrophin complementary DNA (11 kb) and the limited capacity of gene-delivery technologies, such as adeno-associated virus (AAV), which can accommodate up to 4.7 kb. Truncated dystrophins that lack large portions of the protein’s central spectrin-like repeat region have been tested in preclinical studies, where they rescue aspects of the dystrophin-deficient phenotype (7). These miniaturized dystrophin proteins are inspired by naturally occurring dystrophin variants seen in patients that present with a mild form of Becker muscular dystrophy. These patients express dystrophin containing the protein’s N-terminal tandem calponin homology ABD1 (residues 1–246), the first three spectrin-like repeats (R1-R3), and the C-terminal dystroglycan complex–binding region (7). These miniaturized “mini” or “micro” dystrophins can be engineered into an AAV vector, and several are the subjects of ongoing AAV-based gene therapy clinical trials in patients with DMD with the goal of rescuing the linkage between the actin cytoskeleton and the sarcolemmal dystroglycan complex (6).

Dystrophin contains multiple ABDs. The first is ABD1, the N-terminal domain that is present in clinical micro-dystrophin constructs (6, 8). ABD1 is essential for actin binding and for proper protein folding and stability throughout the dystrophin protein (9, 10). The second is ABD2, located within spectrin-like repeats 11 to 17 (8). Both domains contribute to actin binding of full-length dystrophin *in vitro* (11). Removal of ABD2, from dystrophin-based recombinant proteins, results in a 30-fold reduction in actin-binding affinity compared to full-length protein (12, 13, 14). The C-terminal region of dystrophin also influences both actin binding and actin dynamics (14). Micro dystrophin gene therapy constructs lack ABD2 and the C-terminal domain of dystrophin (6), probably resulting in reduced actin-binding affinity *in vivo* as seen in *in vitro* studies. This decreased actin-binding affinity is likely to limit the therapeutic efficacy of these constructs.

Numerous mutations in the ABDs of conserved cytoskeletal dystrophin-related proteins (β-III-spectrin, α-actinin, and filamin) have shown that a small change in the molecular structure of related ABDs can induce dramatic changes in actin-binding affinity (15, 16, 17). We hypothesize that a small-molecule drug can do the same, binding to the WT dystrophin ABD or to actin, inducing similar enhancement of actin-dystrophin affinity. Current *in vitro*–binding assays, such as cosedimentation, are labor-intensive and low-throughput, limited in the number of samples that can be tested and in precision. In the present study, we have developed and validated a novel high-throughput screening (HTS) assay that can detect the binding of actin to dystrophin ABD1, with high specificity, speed, and precision, in the presence and absence of compounds. We have generated a unique fluorescent biosensor, containing ABD1 of WT human dystrophin (hDYS-ABD1) and filamentous actin (Fig. 1). hDYS-ABD1 was fused with a GFP mClover3 (fluorescent donor), while actin was labeled with a fluorescent dye, Alexa Flour 568 (acceptor). We have used a high-precision structural measurement, time-resolved FRET (TR-FRET), to quantitate the binding of donor-labeled hDYS-ABD1 to acceptor-labeled actin (Fig. 1). TR-FRET from hDYS-ABD1 to actin was measured with a recently developed high-speed and high-precision fluorescence lifetime plate reader (FLTPR) (18). Utrophin is an autosomal homolog of dystrophin and has a similar domain structure. Some of the previous actin-binding cosedimentation studies show that the N-terminal ABD1 of utrophin has a tighter affinity for actin than the N-terminal ABD1 of dystrophin (19, 20). This is probably due to the presence of an N-terminal 28 amino acid extension unique to utrophin ABD1. We simultaneously expressed a mClover3-fused human micro-utrophin ABD1 (hUTR-ABD1) construct and used it as a representative of the high-affinity binder. The goal of this study is to identify compounds that can increase the binding affinity of hDYS-ABD1 to actin at least 100 fold, to the level of hUTR-ABD1. We have validated our HTS assay using a 1280-compound library (Library of Pharmacologically Active Compounds, LOPAC) and identified several tool compounds that changed the affinity of actin and hDYS-ABD in a concentration-dependent manner. In search for diverse classes of compounds, we also screened an FDA-approved library (SELLECK). Hits from this HTS assay, defined as compounds producing effects more than 4 SD from the drug-free dimethyl sulfoxide (DMSO) control, were further tested for their efficacy in cosedimentation assays, to evaluate the potential of this TR-FRET approach for drug discovery.

**Figure 1:**
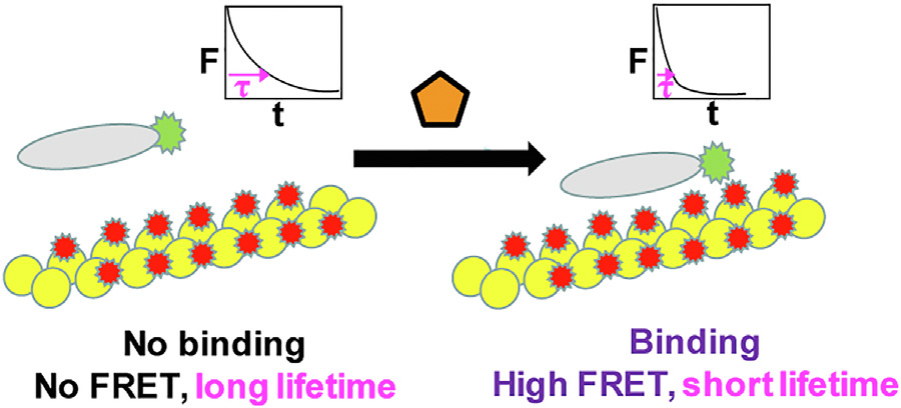
**Dystrophin-actin biosensor, which measures FRET from ABD1-mClover3 (donor, *green star*) to Alexa 568–labeled actin (acceptor, *red star*).** Actin (*yellow*)- or ABD1 (*gray*)-binding compounds (*orange* pentagon) can act as modulators that enhance the binding of ABD1 to actin, as detected by decreased lifetime, providing a potential therapeutic approach to treat DMD. The fluorescent protein mClover3 (*green star*) is fused to the C-terminus of hDYS-ABD1/hUTR-ABD1 (*gray*). ABD, actin-binding domain; DMD, Duchenne muscular dystrophy; hDYS, human dystrophin; hUTR, human utrophin.

## Results

### Biosensor development

**Figure 2:**
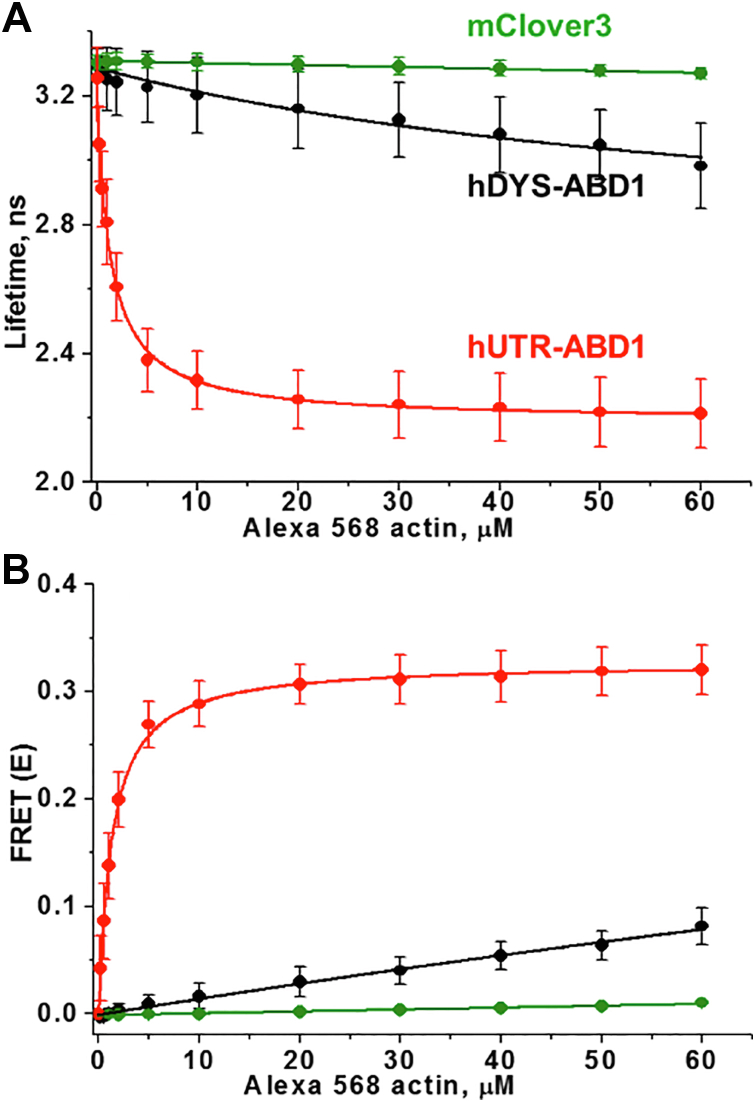
**FRET biosensor detects specific binding of human dystrophin ABD1 (*black*) and human utrophin ABD1 (*red*) to actin in a concentration-dependent manner.***A*, Lifetime and *B*, FRET. Control experiments with mClover3 (*green*), the fluorescent protein without ABD1, show that the FRET change resulted exclusively from actin-ABD1 binding (N = 2–8: errors are SD's for points with N = 3 or greater, while the N = 2 data points are simply means of N = 2). ABD, actin-binding domain.

Time-resolved fluorescence decays of donor-fused hDYS-ABD1 and hUTR-ABD1, in the presence of increasing concentrations of Alexa 568-labeled F-actin (AF-actin), were fitted by a one-exponential decay function using least-squares minimization (see Experimental procedures). The addition of increasing concentrations of AF-actin to hDYS-ABD1 and hUTR-ABD1 decreased the donor lifetime (Fig. 2*A*) and thus increased FRET (Fig. 2*B*). The FRET efficiency (E) was determined as the fractional decrease of donor fluorescence lifetime (τ_D_), due to the presence of acceptor fluorophore (τ_DA_) (Fig. 2). Increased FRET indicates increased actin binding, as confirmed by negligible effects in control FRET experiments with mClover3 construct (no ABD1). Data were evaluated by fitting a single-site hyperbolic-binding model to the actin dependence in FRET. This revealed a kD >100 μM for hDYS-ABD1 and 1.5 ± 1.0 μM for hUTR-ABD1. kD values of hDYS-ABD1 and hUTR-ABD1 binding to actin vary widely, as reported in previous cosedimentation studies (19, 20). The higher affinity of the hUTR-ABD1 construct to actin is most likely attributed to the 28 N-terminal amino acid extensions unique to utrophin’s ABD1, as this flanking N-terminal sequence primarily determines the actin-binding affinity of utrophin’s calponin homology domain (21). The hUTR-ABD1 (compared with hDYS-ABD1) also forms a different structural complex with actin, as shown previously (22). A key advantage of TR-FRET is its ability to detect the bound complex directly, without interference from unbound proteins (23, 24). Thus, TR-FRET, coupled with this biosensor, is able to detect the difference in binding of these two constructs to actin with high-precision, providing sufficient sensitivity for our search for modulators of hDYS-ABD1-actin FRET using HTS.

### HTS assay performance using LOPAC library

**Figure 3:**
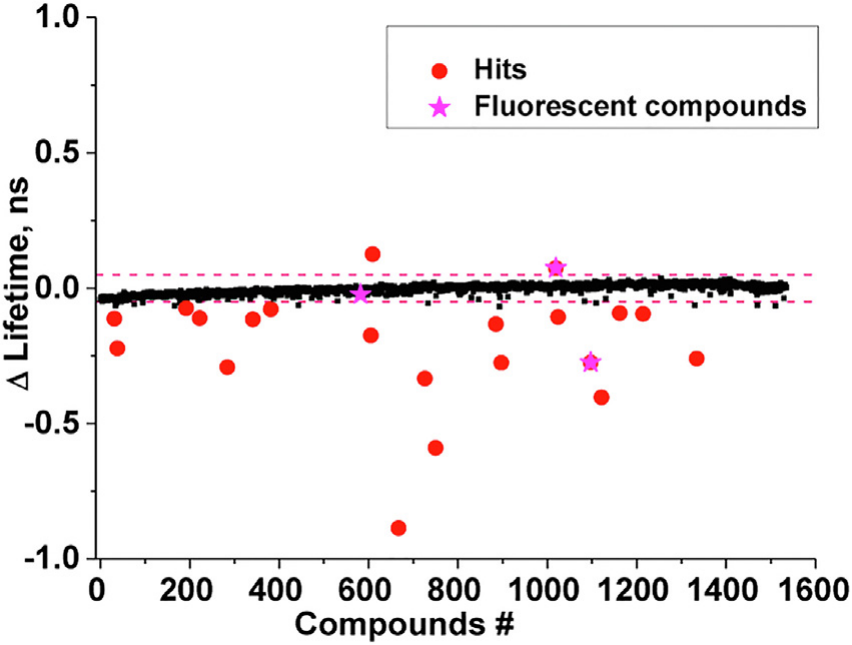
**A representative LOPAC screen.** Hits (*red circles*) were identified as 4SD (showed as *dashed lines*) of DMSO-only control. Interfering (*fluorescent*) compounds were flagged (*magenta*) as they are not real hits. Eleven hit compounds reproducibly changed FRET in triplicate screens. DMSO, dimethyl sulfoxide; LOPAC, Library of Pharmacologically Active Compounds.

Using the FRET biosensor (hDYS-ABD1 plus AF-actin), we performed HTS of the LOPAC library (Fig. 3), a collection of small molecules that have well-documented pharmacological activities. The complete LOPAC library was applied to a single 1536-well black-wall/black-bottom Greiner plate with drug-free control (50 nl of DMSO) wells on each side of the individual plates (see Experimental procedures). For each screen, one LOPAC plate was loaded with 1.0 μM hDYS-ABD1 (donor only), and the other one was loaded with 1.0 μM hDYS-ABD1 and 10 μM AF-actin (donor + acceptor). All plates were incubated for 20 min at 25 °C before reading. Donor hDYS-ABD1 was excited with a 473-nm laser, and time-resolved fluorescence decay waveforms were recorded with the FLTPR. Data was obtained from the entire 1536-well plate within 6 min, after incubation times of 20 and 120 min. The Z′ factor, which validates the robustness of this HTS assay (25), was calculated as 0.8 ± 0.1 using DMSO-only controls. A value above 0.5 indicates a high-quality screen. This screen was performed in triplicate with three different preparations of donor and acceptor samples. A total of 11 compounds reproducibly altered the average lifetime of the donor-acceptor sample by more than 4 SD greater than the mean of the control samples (DMSO). These 11 hit compounds were further tested in concentration–response FRET assays.

### Concentration-response curves using compounds and actin, identification of tool compounds

**Figure 4:**
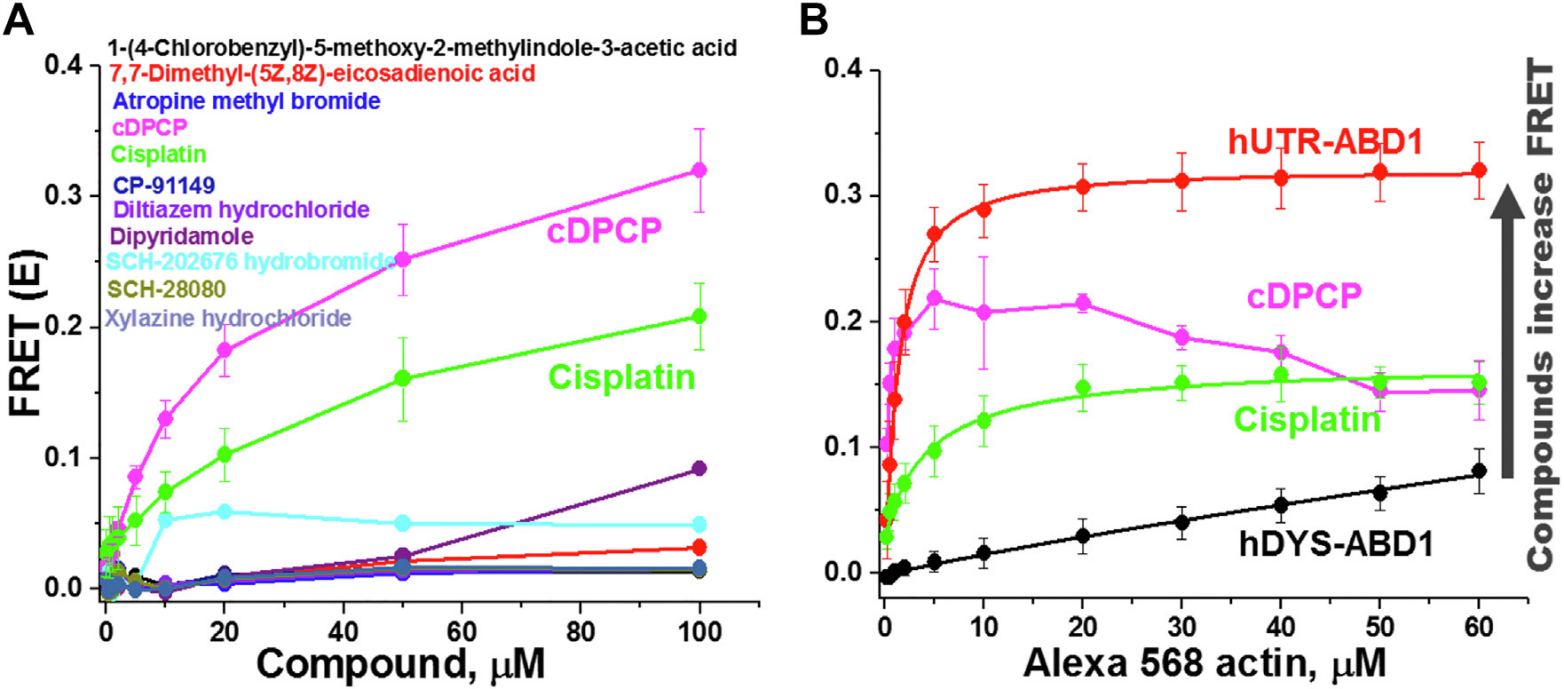
**Concentration-response curves for LOPAC hit compounds.***A*, compound CRC (N = 4–8): 1 μM hDYS-ABD1 and 10 μM AF-actin were incubated with each of the hit compounds (0–100 μM) and FRET was calculated. *B*, actin CRC (N = 3–8): Compounds that had significant effects on CRC were further tested with increasing concentrations of actin. One micromolar hDYS-ABD1 or hUTR-ABD1 was incubated with increasing [AF-actin] using 50 μM compound. Cisplatin and cDPCP both increased the affinity of hDYS-ABD1 for actin, toward the level of hUTR-ABD1. EC_50_ values for Cisplatin and cDPCP are summarized in Table 1. Errors are reported as SD. ABD, actin-binding domain; AF-actin, Alexa 568 F-actin; CRC, concentration-response curve; hDYS, human dystrophin; hUTR, human utrophin; LOPAC, Library of Pharmacologically Active Compounds.

**Figure 5:**
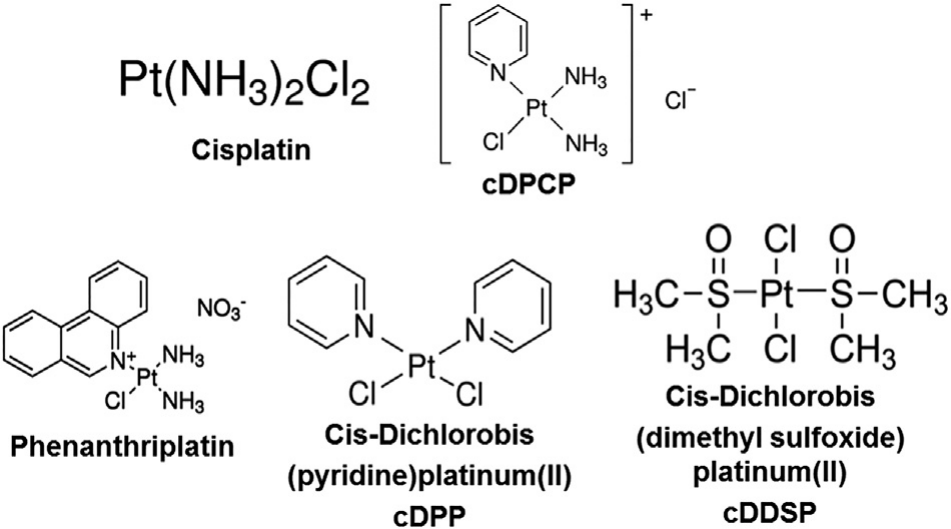
**Cisplatin derivatives.** All four derivatives of cisplatin increase FRET between actin and hDYS-ABD1 in a concentration-dependent manner and have a wide range of EC_50_ (Table 1). These are promising tool/lead compounds for future studies. ABD, actin-binding domain; hDYS, human dystrophin.

Using the same condition as in the primary screen (1 μM hDYS-ABD1 and 10 μM AF-actin), we examined the dependence of FRET on the concentration of each of the reproducible hit compounds (0.5–100 μM). Significant concentration-dependent effects on FRET were observed for several of the identified Hits (Fig. 4*A*). FRET (E) was determined at each compound concentration by the fractional decrease in donor lifetime due to the presence of acceptor. Several of these hit compounds increased FRET at micromolar concentrations with notable decreases in the apparent EC_50_ (concentration needed for half-maximal effect) of the FRET curve (Fig. 4*A*), as desired. Cisplatin, cDPCP ((SP-4-3)-diamminechloro(pyridine)-platinum(1 +), monochloride) and SCH [(-}-trans-6,7,7a,8,9,13b-hexahydro-3-chloro-2-hydroxy-N-methyl- 5H- benzo[d]naptho-(2,1-b)azepine] hydrobromide showed the greatest effects. The effect of these compounds on the hDYS-ABD1 and actin interaction was simultaneously studied with increasing concentrations of actin (Fig. 4*B*) while using a constant concentration of compound (50 μM). While the compound CRC experiment determined the affinity of the compound to either actin or hDYS-ABD1, the actin CRC experiment determined the effect of the compound on the affinity of binding partners. Using the same donor concentration of 1.0 μM, as used in the compound CRC, we varied AF-actin concentration in the range of 0 to 60 μM. Both cisplatin and cDPCP significantly increased the affinity of hDYS-ABD1 for actin (Fig. 4*B*). These two Hits are structural derivatives that contain a platinum (Pt) in their chemical structures (Fig. 5). Other commercially available derivatives of cisplatin, such as phenanthriplatin, cis-Dichlorobis(pyridine)platinum(II), and cis-Dichlorobis(dimethyl sulfoxide)platinum(II), were tested in the compound and actin CRC assays. All of the cisplatin derivatives increased FRET in a concentration-dependent manner, with significant differences in the apparent EC_50_ values (Table 1). Similar effects were observed in the actin-titration assay (Table 1). Thus, using our unique FRET biosensor and HTS assay, we have identified a distinctive class of compounds that can significantly increase the binding between hDYS-ABD1 and actin, as desired.

**Table 1:** EC_50_ (μM) and actin-hDYSABD1 binding kD (μM) of cisplatin derivatives measured by FRET

Compound	EC_50_, μM	E_max_ (FRET)	kD(FRET),μM
No drug			>100
Cisplatin	64.5 ± 7.2	0.33 ± 0.015	4.43 ± 0.8
CDPCP	32.1 ± 9.3	0.43 ± 0.047	0.18 ± 0.07
cis-Dichlorobis(dimethyl sulfoxide)platinum(II)	24.6 ± 9.8	0.17 ± 0.018	5.79 ± 2.5
cis-Dichlorobis(pyridine) platinum(II)	18.7 ± 1.4	0.25 ± 0.008	1.63 ± 0.44
Phenanthriplatin	105.8 ± 33.0	0.37 ± 0.070	n.d.

(N = 2–8, errors are SD's for points with N = 3 or greater, while the N = 2 data points are simply means of N =2).

### Cosedimentation of hDYS-ABD1 and actin using tool compound

**Figure 6:**
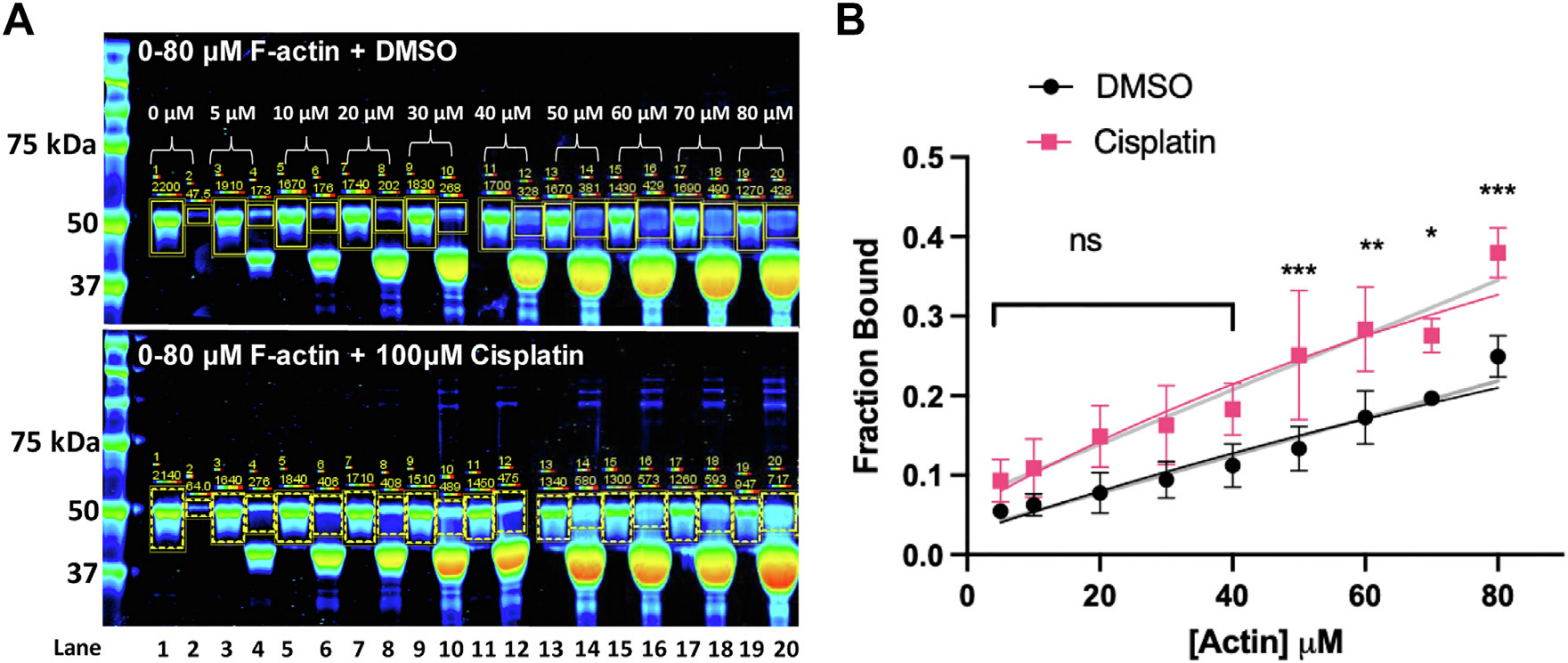
**Cosedimentation.** hDYS-ABD1 (5 μM) was incubated with increasing concentration (0–80 μM) of F-actin at 100 μM cisplatin or 1% DMSO, centrifuged at high speed. *A*, representative cosedimentation SDS-PAGE gel. Odd numbered lanes are supernatant and even numbered lanes are pellet. *B*, bound fraction of hDYS-ABD1 to actin was determined from (A). *Gray lines* indicate linear fit. *Magenta* and *black lines* indicate single hyperbolic fit with Bmax fixed to 1.0 (ns not significant, ∗*p* < 0.0224, ∗∗*p* < 0.0011, ∗∗∗*p* < 0.0005). Errors are reported as SD, (N = 3–4). ABD, actin-binding domain; DMSO, dimethyl sulfoxide; hDYS, human dystrophin.

The effect of cisplatin on the interaction between hDYS-ABD1 and F-actin was examined by cosedimentation of actin and ABD1 (Fig. 6). Five micromolar hDYS-ABD1 was incubated with increasing concentrations of actin (0–80 μM), at a constant concentration (100 μM) of the compound (Fig. 6, magenta). A DMSO control (Fig. 6) was measured simultaneously. Samples were centrifuged at high speed and the bound fraction of hDYS-ABD1 to actin was determined from SDS PAGE, quantifying the amount of protein in the supernatant and pellet using fluorescence imaging and then calculating the fraction of hDYS-ABD1 protein bound to actin. At a constant concentration of the compound, the actin-bound fraction of hDYS-ABD1 was increased with increasing concentrations of actin. Though this increase was not as robust as observed in the FRET experiments, the effect of cisplatin was highly statistically significant for the entire experiment (*p* < 0.0001) and significant for individual actin concentrations above 50 μM determined using a two-way Bonferroni mixed-effect ANOVA test. We fit the actin dependence with a simple linear model and with a single hyperbolic model, with the maximum fraction bound constrained to be not greater than 1.0 (Fig. 6). The slopes of the linear fits were statistically distinct (*p* < 0.01) with cisplatin increasing the slope by 1.5 fold. The kD value estimates from the hyperbolic fits were 1.7 fold different, though the certainty of this estimate is low due to the fact that fraction bound did not reach saturation over the range of actin examined. The statistically significant increase in the fraction of actin-bound hDYS-ABD1 in cisplatin-treated samples provides independent support for the increase in hDYS-ABD1 bound to actin detected by the higher resolution in solution TR-FRET assay. This indicates that TR-FRET HTS screen detected a class of compounds that enhance the actin and hDYS–ABD1 interaction in the expected direction, that is, toward a high-affinity binder, such as hUTR-ABD1.

### Selectivity of the tool compound

**Figure 7:**
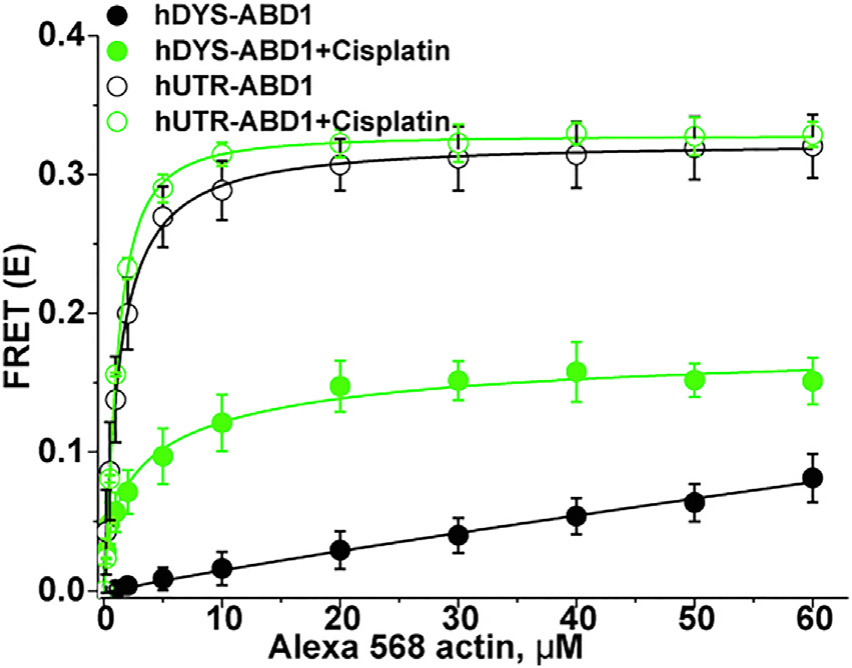
**Selectivity of tool compound (N = 2–8).** Effect of cisplatin is much less on hUTR-ABD1 compared with hDYS-ABD1, indicating that cisplatin acts selectively on actin–DystrophinABD1 complex (N = 2–8: errors are SD's for points with N = 3 or greater, while the N = 2 data points are simply means of N = 2). ABD, actin-binding domain; hDYS, human dystrophin; hUTR, human utrophin.

The effect of cisplatin was also tested on the interaction between actin and hUTR-ABD1. Human utrophin ABD1 binds to actin with a greater affinity than hDYS-ABD1 (Fig. 2). Cisplatin had no significant effect on the binding of hUTR-ABD1 to actin, but it increased the affinity of hDYS-ABD1 to actin by ∼25 fold (Fig. 7), as detected by TR-FRET. This indicates that the identified tool compound specifically enhanced the affinity of hDYS-ABD1 for AF-actin. This is a very promising result, as it shows the potential to identify additional compounds that specifically enhance the interaction between hDYS-ABD1 and actin. These identified compounds will be good candidates for medicinal chemistry to enhance their potency and selectivity.

### Screening of an FDA-approved library

**Figure 8:**
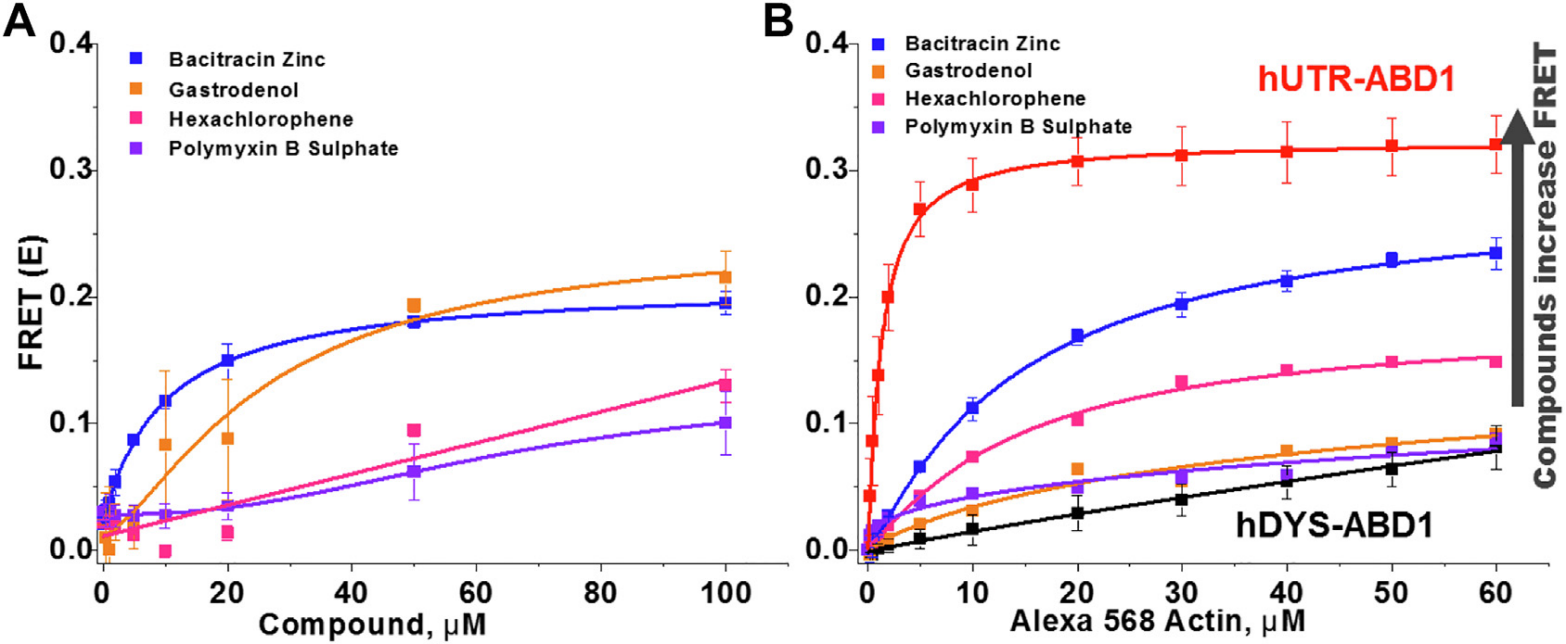
**Concentration-response curves for the promising SELLECK hit compounds.***A*, compound CRC (N = 3): 1 μM hDYS-ABD1 and 10 μM AF-actin were incubated with each of the hit compounds (0–100 μM) and FRET was calculated. *B*, actin CRC (N = 2–8): Compounds were further tested with increasing concentrations of actin. One micromolar hDYS-ABD1 or hUTR-ABD1 was incubated with increasing [AF-actin] using 50 μM compound. All four SELLECK compound increased the affinity of hDYS-ABD1 for actin, toward the level of hUTR-ABD1. EC_50_ values are summarized in Table 2 (errors are SD's for points with N = 3 or greater, while the N = 2 data points are simply means of N = 2). ABD, actin-binding domain; AF-actin, Alexa 568 F-actin; CRC, concentration-response curve; hDYS, human dystrophin; hUTR, human utrophin.

The HTS assay was further used to screen a 2.8 K compound library (SELLECK). This library contains a collection of FDA-approved compounds and allows problematic compounds (*e.g.*, those shown previously to have many nonspecific effects) to be weeded out prior to retests as well as aids in tuning cutoffs for Hit thresholds with flagging for fluorescent compound interference. The SELLECK library was screened twice with independent preparations of actin and hDYS-ABD1. Eighteen reproducible Hits, which increased FRET by at least 4 SD, were further tested in FRET CRC. Of these eighteen compounds, four had the most significant effects on compound CRC and are listed in Figure 8*A* and Table 2. The effects of these four compounds were further examined in actin CRC (Fig. 8*B*). These compounds showed significant increase in FRET in the actin CRC, further validating the sensitivity of the biosensor and the TR-FRET assay.

**Table 2:** EC_50_ (μM) and actin-hDYSABD1 binding kD (μM) of promising SELLECK Hits measured by FRET

Compound	EC_50_, μM	E_max_ (FRET)	kD(FRET),μM
Bacitracin Zinc	9.7 ± 0.2	0.21 ±0.002	14.4 ± 0.8
Gastrodenol	30.4 ± 11.0	0.22 ± 0.04	9.3 ± 1.6
Hexachlorophene	n.d.	n.d.	8.4 ± 1.2
Polymyxin B Sulfate	67.0 ± 14.3	0.13 ± 0.02	5.3 ± 1.3

(N = 2–4: errors are SD's for points with N = 3 or greater, while the N = 2 data points are simply means of N =2).

## Discussion

We report a novel approach to discover a class of compounds that enhance the binding of dystrophin ABD1 to actin, with the ultimate goal of developing improved therapies for muscular dystrophy patients. We have developed a unique FRET biosensor using m-Clover3–fused human dystrophin ABD1 and Alexa 568–labeled filamentous actin and established a HTS assay platform that successfully identified a group of compounds capable of enhancing the actin–hDYS-ABD1 interaction. Each compound’s mechanism of action probably depends on the structure of the compound and its binding site on actin/hDYS-ABD1, resulting in specific changes in the functional interaction of actin and hDYS-ABD1.

Gene therapy strategies to replace dysfunctional dystrophin are the most promising treatment options currently available for DMD patients (6). However, these gene therapy constructs do not contain all the domains of dystrophin that are required for efficient actin binding, so actin binding of these constructs is likely to be weaker than that of full-length dystrophin. The binding affinities of hDYS-ABD1 and hUTR-ABD1 to actin were previously measured using cosedimentation, either varying actin or varying dystrophin, and kD values reported have been somewhat inconsistent (20, 26, 27). The FRET-biosensor developed here is capable of detecting actin and hDYS-ABD1 binding with high accuracy, precision, and speed. Previously, pyrene iodoacetamide–labeled actin has been widely used to monitor the binding of actin-binding proteins (ABPs), like myosins (24, 28). Pyrene-labeled actin needs to be excited at 355 nm and can only detect the strongly bound actomyosin complex. A large number of small molecules are excited at 355 nm and can generate background fluorescence. The advantage of using Alexa 568–labeled actin is that it is more red-shifted and is free of background fluorescence from most compounds. This FRET sensor is also sensitive enough to differentiate between the interaction of actin and a low-affinity binder (hDYS-ABD1), a high-affinity binder (hUTR-ABD1), and a nonbinder protein (mClover3) (Fig. 2). This approach is distinct compared to our previous TR-FRET approaches for studying ABPs such as myosin (29), β-III spectrin (30), or myosin binding protein C (31). Previously, we utilized small peptides (29), unlabeled ABP (31), or cell-based assays for monitoring the interaction between actin and a specific ABP. The FRET-biosensor used here is advantageous as it is utilizing the actual dystrophin or utrophin ABD fused with a fluorescent protein and fully labeled actin and is also free of any nonspecific FRET signal that is characteristic of the cell-based assays. Thus, the current approach is more sensitive, which is also reflected in the cosedimentation measurement, a nonfluorescent-binding assay. In the present study, compounds identified from the LOPAC or SELLECK libraries that altered the interaction between actin and hDYS-ABD1 were also detected with high-precision. The optimized HTS assay using this sensor showed a Z′ value of 0.8 ± 0.1 in 1536 well format, which is considered excellent in the field of HTS assay development (32). Our primary HTS assay identified several compounds that significantly enhance the affinity of the actin–hDYS-ABD1 complex. These compounds could bind either to actin or to hDYS-ABD1. Compounds that affect the donor (hDYS-ABD1) lifetime in the absence of acceptor probably bind to hDYS-ABD1 and alter the environment of fused mClover3. On the other hand, compounds that have negligible effects on the donor-only lifetime but increase the overall FRET probably bind to actin or to hDYS-ABD1 in a way that does not affect the lifetime of the attached mClover3 donor.

**Figure 9:**
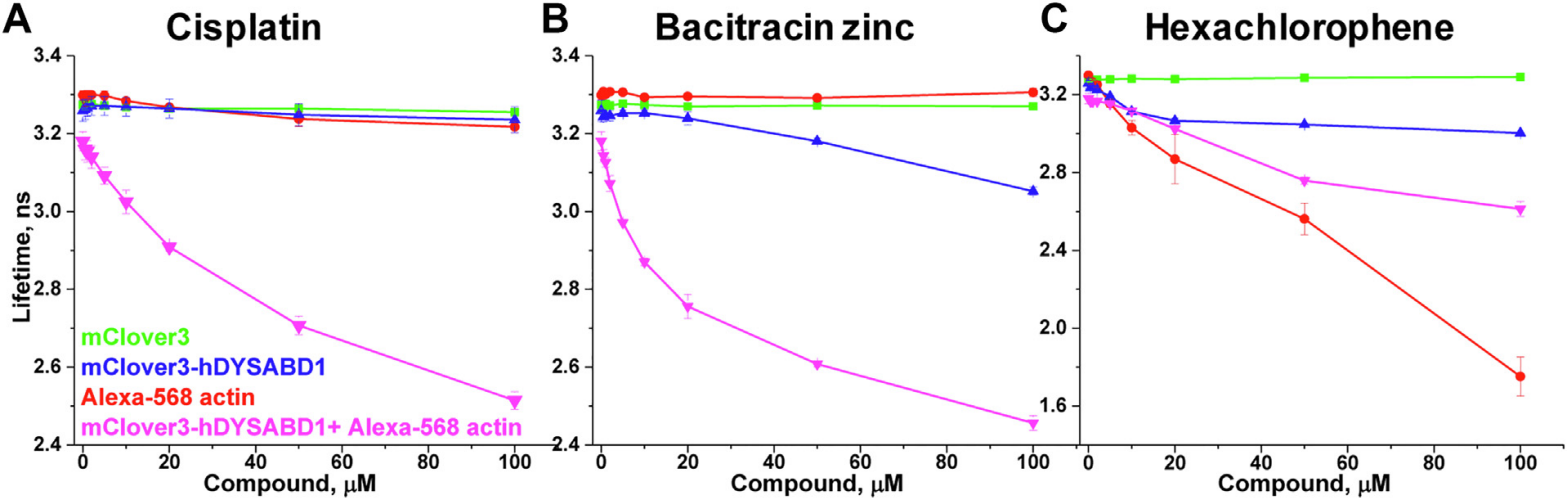
**Concentration response effects of the most promising compounds on mClover3 (fluorescent protein, no ABD1,*green*), mClover3-ABD1 (Donor only, *blue*), mClover3-ABD1 +Alexa 568 actin (Donor + Acceptor, *magenta*), and Alexa 568 actin (acceptor only, *red*).** Fluorescent protein control, donor, and donor+acceptor samples were excited at 473 nm and acceptor only sample was excited at 532 nm. *A*, Cisplatin. *B*, Bacitracin Zinc. *C*, Hexachlorophene. (N = 4: Errors are reported as SD). ABD, actin-binding domain.

Control experiments (Fig. 9) on the three most promising compounds (cisplatin, bacitracin zinc, and hexachlorophene) clearly indicate that none of these compounds affect the fluorescence of mClover3. Therefore, the effects of these compounds on the structural complex are either due to their binding to actin or hDYS-ABD1 or both. Excitation of the donor-only sample (hDYS-ABD1) at 473 nm or the acceptor-only sample (AF actin) at 532 nm shows that the mechanisms of actions of these three compounds are different. For cisplatin, the effect is more pronounced on the two-protein complex than on the individual protein (Fig. 9*A*), whereas for bacitracin zinc (Fig. 9*B*), the effect is mainly due to the change in the donor-only sample. Hexachlorophene affects both of the binding partners (Fig. 9*C*). Actin polymerization measurements can confirm the impact of these compounds on the G- to F-actin transition and on actin filament dynamics (29, 33).

Small-molecule effectors have already been a part of the therapeutic strategies for DMD, specifically to target the downstream effects. These small molecules are targeting DMD-associated pathological changes, such as inflammation, fibrosis, muscle damage, oxidative stress, muscle ischemia, muscle atrophy, and bone homeostasis and are in different phases of clinical trials (5). Our approach is distinctive in that it focuses on improving the existing gene therapy procedures with the intervention of appropriate compounds. The main group of compounds identified from the LOPAC library is based on cisplatin, a well-known chemotherapeutic agent that induces apoptosis in cancer cells (34). As shown in Figure 5, all cisplatin derivatives contain a Pt ion at the center. From the molecular perspective, cisplatin represents a good example of how a small alteration in chemical structure can significantly affect biological activity in a target cell (35). We see a similar effect here. Cisplatin and its derivatives showed a wide range of effects on actin and hDYS–ABD1 interactions (Table 1). While cisplatin has a significant effect on actin–hDYS-ABD1 interaction, its influence on the actin–hUTR-ABD1 interaction is negligibly small (Fig. 7). This is promising, as it shows that the modification of structures with medicinal chemistry could potentially lead to the generation of new scaffolds with reduced side effects and toxicity and increased specificity for dystrophic muscle pathology. Bacitracin zinc, gastrodenol, hexachlorophene, and polymyxin-B-sulfate (Table 2) from the SELLECK library showed significant concentration-dependent increased FRET between actin and hDYS-ABD1. These compounds are already used as antimicrobial and antiseptic agents. Bacitracin and gastrodenol also contain a heavy metal at the center of their structure, as does cisplatin. The main goal of our approach is to identify a molecular scaffold that can further be developed into an improved therapeutic for DMD. Notably, rational design approaches, including pursuit of structural analogs of the identified compounds and *in silico* docking, may identify additional candidate small molecules. The main challenge will be to see the effects of these compounds in *mdx* mice, whether they can maintain a balance in the actin and micro-dystrophin binding that is optimum enough to perform cellular functions without the large side effects. We aim to apply the optimized assay and protocols reported here to screen larger libraries with greater diversity in molecular scaffolds, to identify small molecules that directly bind either to actin or to ABD and increase the actin affinity of micro-dystrophin to the level of full-length dystrophin. Identified compounds from the LOPAC and SELLECK libraries are toxic and not suited for the chronic dosing required for DMD treatment and thus require further exploration by medicinal chemistry.

Mutations in the ABDs of other conserved dystrophin-related cytoskeletal proteins (*e.g.*, β-III-spectrin, α-actinin, and filamin) have shown that small changes in the aa sequences of related ABDs can induce dramatic changes in their actin-binding affinity and cause life-threatening disorders (15, 16, 17). The FRET-biosensor and the HTS assay platform reported here have the potential to identify a unique molecular scaffold for DMD and can also be adapted to study these other actin-linked cytoskeletal disorders. Overall, our FRET-based HTS platform sets the stage to screen large compound libraries for modulators of actin-dystrophin, or disease-linked dystrophin-related proteins, for therapeutic development.

## Experimental procedures

### Expression and purification of ABD1 constructs

The hDYS-ABD1-mClover3 and hUTR-ABD1-mClover3 were cloned into the pDT68 plasmid, a pET-derived expression plasmid containing an N-terminal 6His tag followed by a Sumo tag (36) vector. pDT68 was a gift from Dr Wendy Gordon at the University of Minnesota. The fluorescent protein mClover3 was fused at the C-terminal of both of the ABD1 fragments. Sequence-verified expression plasmids were transformed into the BL21 *E*. *coli* strain and were expressed by IPTG induction for 3 h at 37 °C. Cells were then harvested by centrifugation, and the harvested pellets were resuspended in the lysis buffer. Cells were further disrupted by sonication in the presence of protease inhibitors and subjected to gravity flow-based NiNTA affinity purification using a resin purchased from Thermo Fisher Scientific. The purified protein was dialyzed overnight into PBS with 1 mM DTT in the presence of the ULP1 Sumo-tag protease (Sigma Aldrich). The cleaved 6His-Sumo-tag was removed by size-exclusion chromatography. The mClover3 protein was expressed and purified in a similar way and was used as a control in FRET and cosedimentation experiments. Protein concentration was measured using nanodrop, and protein was snap frozen in liquid Nitrogen in PBS with 1 mM DTT and stored at −80 °C.

### Actin preparation and labeling

Actin was prepared from rabbit skeletal muscle by extracting acetone powder in cold water, as described previously (37). One hundred thirty micromolar Alexa Fluor 568 C5 maleimide (Invitrogen), freshly dissolved in dimethylformamide, was added to 65 μM F-actin and the sample was incubated for 30 min at 25 °C and then for 18 h at 4 °C. Labeling was terminated by adding 10 mM DTT, and actin was ultracentrifuged for 30 min at 350,000*g*. The F-actin pellet was suspended in G-Mg buffer (5 mM Tris, 0.5 mM ATP, 0.2 mM MgCl_2_, pH 7.5) followed by clarification at 300,000*g* for 10 min. Actin was again polymerized for 45 min at 25 °C in the presence of 3 mM MgCl_2_ and ultracentrifuged at 350,000*g* for 30 min. F-actin pellet was suspended in F-Mg buffer (3 mM MgCl_2_, 10 mM Tris, pH 7.5) containing 0.2 mM ATP. The labeled F-actin was immediately stabilized against depolymerization and denaturation by adding equimolar phalloidin.

### Fluorescence data acquisition

Fluorescence lifetime measurements were carried out by a high-precision FLTPR (Photonic Pharma LLC) (18, 38). Donor samples (mClover3, mClover3 hDYS-ABD1, and mClover3 hUTR-ABD1) were excited with a 473-nm microchip laser (Bright Solutions), and emission was filtered with 488-nm long pass and 517/20-nm band pass filters (Semrock). This instrument enables high-throughput fluorescence lifetime detection at high precision by utilizing a unique direct waveform recording technology (38). The performance of this FLTPR has been previously demonstrated with FRET-based HTS that targets several muscle and nonmuscle proteins (30, 38, 39). In the present study, modifications were made in the instrument to permit 2-channel detection, for the purpose of flagging false Hits due to interference from fluorescent compounds.

### Screen with LOPAC and SELLECK library

The 1280 LOPAC and 2800 SELLECK compounds were received in 96-well plates and reformatted into 1536-well flat, black-bottom polypropylene plates (Greiner Bio-One). In total, 50 nl of each compound solution was dispensed in DMSO using an automated Echo 550 acoustic liquid dispenser (Labcyte). Compounds were formatted into the assay plates, at a final concentration of 10 μM, with the first two and last two columns loaded with DMSO only (compound-free controls). These assay plates were then heat-sealed using a PlateLoc Thermal Microplate Sealer (Agilent Technologies) and stored at −20 °C. Before screening, compound plates were equilibrated to room temperature (25 °C). In total, 1.0 μM mClover3/hDYS-ABD1/hUTR-ABD1 without or with 10 μM Alexa 568–labeled actin was dispensed by a Multidrop Combi Reagent Dispenser (Thermo Fisher Scientific) into the 1536-well assay plates containing the compounds. Plates were incubated at room temperature for 20 min before recording the data with the FLTPR. Plates were rescanned after 120 min incubation.

### HTS data analysis

Waveforms for each well in HTS were convolved with the instrument response function and were fitted by a one-exponential decay function using least-squares minimization (40). The FRET efficiency E was determined as the fractional decrease in donor fluorescence lifetime (τ_D_) due to the acceptor (τ_DA_). Assay quality was determined based on FRET assay samples in wells preloaded with control (DMSO) and tested tool compound, as indexed by the Z′ factor: a value of 0.5 or higher indicating excellent assay quality

$$Z^{\prime }=1-3\left[\left(\sigma_{DMSO}+\sigma_{Tool}\right)/\left(\mu_{DMSO}-\mu_{Tool}\right)\right]$$

where σ_DMSO_ and σ_Tool_ are the SDs of the DMSO τ_DA_ and tool compound τ_DA_, respectively; μ_DMSO_ and μ_Tool_ are the means of the DMSO τ_DA_ and tool compound τ_DA_, respectively. A compound was considered a Hit if it changed τ_DA_ by >4SD relative to that of control τ_DA_ that were exposed to 0.1% DMSO.

### Compound’s concentration–response assay

The Hit compounds were purchased and dissolved in DMSO to make a 10 mM stock solution, which was serially diluted in 96-well mother plates. Hits were screened at eight concentrations (0.5–100 μM). Compounds (1 μl) were transferred from the mother plates into 384-well plates using a Mosquito HV liquid handler (TTP Labtech Ltd). The same procedure of dispensing as for the pilot screening was applied in the TR-FRET concentration–response assays. The FRET efficiency E was determined as the fractional decrease in donor fluorescence lifetime as described above. Concentration dependence of the TR-FRET change was fitted using the Hill equation:

$$\tau =\tau_{0}+\left[\tau_{\max}C^{\alpha }/\left(E{C_{50}}^{\alpha }{+C}^{\alpha }\right)\right]$$

where τ and τ_0_ are TR-F in the presence and in the absence of the compound, τ_max_ is the maximum effect, *C* is the compound concentration, EC_50_ is the compound concentration for which 50% of maximum effect is obtained, and α is the Hill coefficient of sigmoidicity. Selected compounds were also tested for their binding specificity to mClover3 (no ABD1) or hDYS-ABD1 by exciting the sample at 473 nm. In addition, binding specificity of the selected compounds to actin were examined by exciting the acceptor-only sample (AF-actin) at 532 nm using a different micro-chip laser, and emission was filtered with 532- and 573-nm dichroic long pass filters and 546- and 586-nm band pass filters.

### Actin concentration–response assay

This assay was performed using 1.0 μM mClover3/hDYS- ABD1/hUTR-ABD1, with increasing concentrations of AF-actin (0–60 μM) at a constant concentration of 50 μM compound. The FRET efficiency E at each concentration of actin was determined as described above, and kD of binding was calculated from fitting data into a hyperbolic model.

### Cosedimentation

The binding of F-actin and hDYS-ABD1 was performed by cosedimentation as described previously (12), with minor modifications. Before assay, hDYS-ABD1 was clarified at 50K for 30 min. Five micromolar of hDYS-ABD1 was incubated with increasing concentrations of phalloidin-stabilized F-actin (0–80 μM) with or without 100 μM of compound, for a total reaction volume of 60 μl in F-buffer containing 10 mM Tris, pH 7.5, 50 mM KCl, 0.2 mM ATP, 3 mM MgCl_2_, and 0.5 mM DTT. hDYS-ABD1 and compound concentration were chosen according to the sensitivity of the cosedimentation assay. Samples were incubated at 25 °C for 30 min, then centrifuged at 50K for 30 min. The fraction of ABD1 bound to actin was quantified using SDS-PAGE and Coomassie blue staining (13). Compound effect on ABD1 aggregation was measured using a control sample that lacked actin. The data was fitted with a simple linear model and with a single hyperbolic model, with the maximum fraction bound constrained to be not greater than 1.0. Statistical analysis was performed using 2-way Bonferroni mixed-effect ANOVA test (Fig. 6).

#### Error analysis

All errors are reported as mean ± SD.

## Data availability

All data discussed are presented within the article.

## Conflict of interest

D. D. T. holds equity in and serves as President of Photonic Pharma LLC. This relationship has been reviewed and managed by the University of Minnesota. Photonic Pharma had no role in this study, except to provide instrumentation, as stated in Experimental procedures. J. M. M., J. M. E., and D. D. T. are entitled to royalties from Sarepta Therapeutics, the company sponsoring this research project. This royalty interest has been reviewed and managed according to the University of Minnesota's conflict of interest policies. J. M. E. has received compensation for consulting for Sarepta. This relationship has been reviewed and managed by the University of Minnesota in accordance with its conflict of interest polices. All other authors declare no conflicts of interest with the contents of the article.
